# Evaluation of a Manual Cytocentrifuge versus the Standard Automated Cytocentrifuge in the Analysis of Canine Cerebrospinal Fluid: A Case Series of 55 Dogs

**DOI:** 10.3390/vetsci10110631

**Published:** 2023-10-24

**Authors:** Luísa Fonte-Oliveira, André Pereira, Hugo Gregório, João Ribeiro, Carla Correia-Gomes, Ricardo Marcos, Marta Santos

**Affiliations:** 1 Cytology and Hematology Diagnostic Services, Laboratory of Histology and Embryology, Department of Microscopy, ICBAS-UP, School of Medicine and Biomedical Sciences, Universidade do Porto, Rua de Jorge Viterbo Ferreira, 228, 4050-313 Porto, Portugalrmarcos@icbas.up.pt (R.M.); 2 AniCura CHV Porto Hospital Veterinário, 4100-320 Porto, Portugal; andre.pereira@anicura.pt (A.P.); hugo.gregorio@anicura.pt (H.G.); 3 Referência Veterinária, 2645-550 Alcabideche, Portugal; neurovet@outlook.pt; 4 Faculdade de Medicina Veterinária, Universidade Lusófona de Humanidades e Tecnologias, 1749-024 Lisboa, Portugal; 5 Animal Health Ireland, Carrick on Shannon Co., N41 WN27 Leitrim, Ireland; carlasofiagomes@gmail.com

**Keywords:** cytology, cerebrospinal fluid, dog, manual cytocentrifugation, cytospins, pleocytosis

## Abstract

**Simple Summary:**

Veterinary cytology has become a cornerstone method for confirming initial clinical diagnosis, guiding early owner communication and therapeutic planning. Cytology is often acclaimed for the ease of obtaining samples, quick results and lower cost when compared with other diagnostic tests. The analysis of cerebrospinal fluid plays a central role in the management of neurologic patients. Cerebrospinal fluid is typically a low-protein, low-cell fluid that must be rapidly cytocentrifuged to obtain a good-quality cytologic slide, called a cytospin. Automated cytocentrifuges are relatively expensive pieces of equipment typically only available in veterinary and human diagnostic laboratories, but are not usually available for use in routine clinical practice. A low-cost manual cytocentrifuge is readily available and has been developed to obtain in-clinic cytospins of a variety of fluid samples. In this study, the use of a manual cytocentrifuge was validated for analysis of canine cerebrospinal fluids in routine clinical practice.

**Abstract:**

Cytospins are important for evaluating fluids with very low cellularity such as cerebrospinal fluid (CSF). The aim of this study was to compare the CSF cytospin preparations obtained from automated and manual cytocentrifugation methods. A prospective case series was performed to analyze canine CSF samples using both centrifugation methods. The cytospins were processed within 30–60 min and prepared simultaneously in a conventional automated cytocentrifuge and in an in-house manual cytocentrifuge, using a fixed volume of CSF fluid. The cellularity, differential cell count and the proportion of cell artifacts (pseudopods and vacuolization) were blindly assessed in the cytospin preparations obtained using the two methods. The agreement and correlation between both methods were analyzed. There were 55 dogs enrolled (48 prospectively and 7 retrospectively) in the study. 38 dogs had normal total nucleated cell counts, while 17 had pleocytosis. Automated and manual cytocentrifugation had similar cell yields, and no significant differences in differential cell counts or the presence of artifacts existed between both methods. In cases with pleocytosis, the cytologic diagnosis obtained using each method was similar. Manual cytocentrifugation of CSF is a reliable and economic method designed for routine clinical practice. Its use reduces the specimen deterioration related to processing and analysis delays when samples are transported to external laboratories for evaluation.

## 1. Introduction

The evaluation of CSF plays a central role in the management of canine neurologic patients [1,2]. In contrast with human medicine, biopsies of the brain and spinal cord are rarely performed in dogs and cats. The analysis of CSF is laborious and time-consuming and its analysis is more accurate when performed immediately after collection. Routine analysis of CSF includes the following: (1) macroscopic evaluation; (2) quantitative analysis (erythrocyte count, total nucleated cell count (TNCC), differential cell count and total protein) and (3) microscopic cytologic evaluation [3,4]. CSF evaluation can identify abnormal changes and, in combination with other tests, help narrow the spectrum of differential diagnosis or even yield a specific diagnosis. Therefore, CSF analysis has high sensitivity, but low specificity for the detection of disease [1,3,5]. For these reasons, an accurate history, physical and neurological examinations, imaging studies and other diagnostic tests are essential for an accurate and correct interpretation of the CSF changes in an individual case [5]. 

The erythrocyte (RBC) count and TNCC are often measured using standard manual hemocytometers, such as a Neubauer counting chamber [3,4,5,6,7]. Manual counts continue to be superior to the automatic counts performed in hematology analyzers in CSF specimens with a low TNCC [6,8]. Practice is needed to differentiate erythrocytes from white blood cells (WBCs), especially small mature lymphocytes, because of their similar size. When an unstained specimen is examined, the RBCs lack a nucleus and internal structure and the WBCs are larger and have a granular appearance. Alternatively, the nucleus of the WBCs may be stained with a small amount of new methylene blue stain whilst in a microhematocrit tube, and the stained CSF can be drawn into the hemocytometer chamber [4,5]. 

In relation to microscopic evaluation, because CSF has low cellularity, a concentration procedure is generally required. Different methods of cytologic preparation have been described for the qualitative analysis of CSF in clinical practice. Many of these methods are complicated, making their use in clinical practice difficult [9]. Various inexpensive cell concentration techniques have been proposed, including membrane filtration [10], sedimentation chambers [3,9,10,11], the sedimentation of CSF direct from the needle [12], the line smear technique [13] and manual cytocentrifugation [14]. The most common techniques are cytocentrifugation and sedimentation [3,9,10,11]. The smears prepared using cytocentrifugation (so-called cytospins) are widely used in human and veterinary cytology. However, the relatively high cost of commercial cytospin centrifuges has limited their availability in veterinary clinics and some clinical laboratories [9,14]. Sedimentation techniques are more suitable for use in practice when rapid laboratory submission of CSF specimens is not possible. Despite being an accessible and unexpensive in-house technique, the CSF fluid added to the sedimentation chamber must sit undisturbed for one hour, which can negatively affect the quality of cell morphology [3]. Delayed processing can result in nuclear pyknosis, lysis and disintegration of the cytoplasmatic and nuclear membranes [5]. Additionally, cytocentrifugation can induce artifacts and morphologic alterations in cells, such as vacuolization, increase in cell volume and nuclear irregularities [15]. These artifacts may complicate the classification of mononuclear cells in CSF based solely on microscopic appearance [15].

In 2016, Marcos et al. described a quick, simple and affordable manual method to concentrate low-cellularity fluids. Besides having a low cost, as is generally demanded in frugal science [16], the manual cytocentrifuge produced cytospins with a similar cell yield to the automated cytocentrifuge in a restricted number of samples [14]. It also allowed for immediate CSF analysis. Thus, the manual cytocentrifuge method may be a valuable option for routine CSF evaluation in a clinical practice setting. A prospective comparison of the use of a manual cytocentrifuge with CSF compared to the standard automated cytocentrifuge has been missing in the literature. 

The aim of this study was to prospectively compare the performance of a manual cytocentrifuge with that of an automated cytocentrifuge for use in canine CSF analysis. The cell yield, differential cell counts and the presence of artifacts in cytologic samples prepared by both methods will be compared. 

## 2. Materials and Methods

### 2.1. Sample Collection and Case Selection

A prospective study was designed, including a systematic sample of dogs of any age, breed or sex that presented to AniCura CHV Porto Hospital Veterinário and to Referência Veterinária (inclusion of cases was performed during a specific week) and were anesthetized for computed tomography and/or magnetic resonance imaging of the brain and for spinal CSF tap. For study inclusion, each owner provided informed and written consent. Comprehensive generalized bloodwork, including a cell blood count and biochemistry panel, was performed for every patient prior to anesthesia. The CSF was collected from the cerebellomedullary cistern (also known as the cisterna magna) or the caudal lumbar subarachnoid space, following state-of-the-art procedures being carried out by a veterinary neurologist. In all the cases, CSF analysis was performed immediately after collection, within 30 to 60 min after collection [4,8]. Furthermore, archived canine CSF cytospins cases were retrospectively selected from the archives of the Cytology and Hematology Diagnostic Services at ICBAS, University of Porto, Portugal. These cases had been included in a smaller case study to validate the use of a manual cytocentrifuge for different types of specimens with low cellularity [14], so the smears had been prepared following the same rules as the prospective cases.

### 2.2. Cell Counts

In each CSF specimen, the TNCC and RBC count were measured separately using a manual hemocytometer (Neubauer counting chamber), by using the new methylene blue stain to differentiate the nucleated cells (monocytoid cells, lymphocytes and polymorphonuclear) from the erythrocytes. The cells were stained with a small amount of stain whilst in a microhematocrit tube and, after 5 to 10 min, the stained CSF was drawn into the hemocytometer chamber [5].The TNCC and the RBCs were counted separately. A normal TNCC was considered if fewer than 5 cells/μL were counted and cell counts higher than 5 cells/μL were considered a case of pleocytosis [4]. Pleocytosis was considered mild, moderate or severe if 6 to 50 cells/μL, 51 to 1000 cells/μL or more than 1000 cells/μL were present, respectively [4]. 

### 2.3. Cytocentrifugation

**Figure 1:**
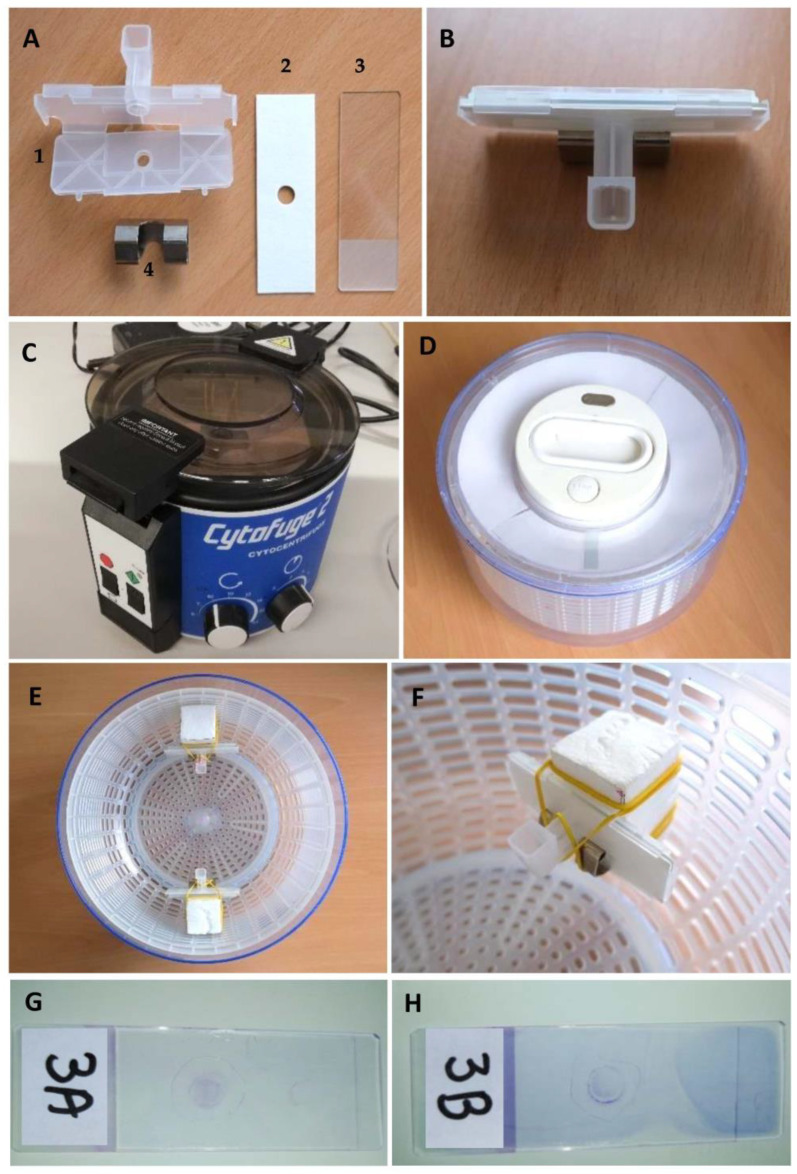
Cytocentrifugation material preparation: reusable cell concentrator (**A1**), disposable filter (**A2**), glass slide (**A3**) and metallic clip (**A4**), appropriately assembled for cytocentrifugation (**B**), with automatic (**C**) and manual (**D**) cytocentrifuges. In the latter, Styrofoam cushions were attached in equidistant positions (**E**), using rubber bands to hold the cell concentrator (**F**). Random number designation of cases and random A/B labeling of the slides was performed; this corresponded to the automated cytospin (**G**) and the manual cytospin (**H**) of case number 3.

In each case, two cytospins were prepared simultaneously using the manual and automated cytocentrifuge, following the instructions described previously [14]. In both procedures, an equal volume (from 100 to 200 μL, depending on the total collected volume in each dog) of CSF was pipetted into a reusable cell concentrator (VWR cat: 720-1972; CytoSep, Beloeil, QC, Canada) attached to a glass slide layered with a disposable filter with a central hole 7.25 mm in diameter (VWR cat: 720-1973), and held together with clips (StatSpin cat: FFCL) (Figure 1A,B). The CSF specimen was then cytocentrifuged in a Statspin Cytofuge 2 (Figure 1C) at 850 rpm for 8 min, following the manufacturer’s recommendations. In relation to the manual cytocentrifuge, the CSF specimen was placed on a modified commercial salad spinner (26 cm diameter, Zyliss cat: 15021; Diethelm Keller brands, Zurich, Switzerland) (Figure 1D). This device was accelerated by pulling a handle continuously to half the distance of the string. Styrofoam cushions of 5 × 3 × 2 cm were fitted to the basket in an equidistant position (Figure 1E) with rubber bands, which also kept the cell concentrators in place (Figure 1F). On average, the basket rotated at 850–1150 rpm as measured using a digital tachometer (DT-2234C; Rinch Industrial, Shangai, China, accuracy ± 1 rpm) for 5 min, following the previous instructions [14,17]. All slides were stained at the same time using a Romanowsky-type stain (Hemacolor; Merck, Darmstad, Germany) and mounted with mounting media (Coverquick 2000; VWR Chemicals, Fontenay-sous-Bois, France). A randomly designated number of cases and random A/B labeling of slides were used to assure a blinded cytological evaluation of each cytospin (Figure 1G,H).

### 2.4. Microscopic Evaluation 

The cytological evaluation was blindly performed in both cytospins by the same observer (LFO). The cellularity was obtained by counting all the WBCs from the slide (if less than 200 cells were present) or 200 cells in samples with pleocytosis. In the same way, differential cell counts distinguishing monocytoid cells from lymphocytes, neutrophils or others, on stained cytospin smears, were performed manually in 200 cells or in all the cells if the cytospins presented fewer than 200 cells [4,8] in the paired cytospin smears, in all cases. Equally, the erythrocyte count and the number of non-diagnostic cells were also blindly assessed in both cytospins. 

### 2.5. Pleocytosis Classification

The pleocytosis was classified according to the cell type that comprised 70% or more of the nucleated cell population (neutrophils, lymphocytes or monocytoid cells). A mixed-cell pleocytosis was determined when the major cell type percentage was 50% or less. In cases where the predominant cell type proportion was between 50% and 70%, the pleocytosis was also classified as mixed-cell, but mentioning the predominant cell type [4]. If eosinophils corresponded to 10 to 20% of the nucleated cell population, the pleocytosis would be classified as eosinophilic [4]. 

### 2.6. Presence of Artifacts 

Cytocentrifuged cells tend to appear larger and artifacts may develop, such as cytoplasmatic vacuoles or pseudopods [4,15,17]. In this study, the presence and the number of cells presenting these two types of artifacts were recorded in the prospective cases. 

### 2.7. Statistical Analysis

The RStudio 2023.06.1 software (Posit Software, PBC, Boston, MA, USA) was used. The normality of the variables was tested using the Shapiro–Wilk test. The differences between the two methods in terms of the total of nucleated cells, % of monocytes, % of neutrophils, % of lymphocytes, % of non-diagnostic cells, % vacuoles, % pseudopods and RBCs were assessed using the Wilcoxon signed rank test. The agreement between the two methods in terms of total cells and the presence of artifacts and RBCs was assessed with kappa statistics using the package psych [18]. For interpreting the strength of agreement, the following standards were considered: 0.40 poor, 0.41 to 0.60 moderate, 0.61 to 0.80 good and 0.81 to 1 almost perfect. Spearman’s correlation test was used to test the correlation between the methods according to several variables. A *p* < 0.05 was considered to define statistical significance.

## 3. Results

### 3.1. Study Case Series

From April 2021 to May 2023, 48 cases were prospectively enrolled, belonging to dogs aged between 18 months and 14 years of age (30 males and 18 females). Additionally, 7 retrospective cases of CSF collected from January to December 2015 from dogs aged between five months and nine years of age (5 males and 2 females) were also included. 

In this case series, 38 out of 55 cases presented with a TNCC ≤ 5 cells/μL (defined as not having pleocytosis), while 17 out of 55 cases presented a TNCC > 5 cells/μL (pleocytosis), 8 of which had mild, 5 moderate and 4 severe pleocytosis (Table 1). 

### 3.2. Cytocentrifugation Technique

Both cytospins were easily processed at the same time, while the CSF in the microhematocrit tube was stained using new methylene blue for cell counting in the Neubauer chamber. The same pace and strength were used when pulling the handle during manual centrifugation for a 5 min duration. This procedure was carried out simultaneously with the automatic cytocentrifugation and did not jeopardize or delay the CSF evaluation. 

In all the cases, the smears obtained using both devices resulted macroscopically in good-quality cytospins, with cells distributed over the circular area in the center of the slide (Figure 1G,H). 

### 3.3. Comparison of Cell Counts and WBCs Differential 

In one case, the automatic cytospin did not render any cells, whilst five cells were observed in the corresponding manual cytospin. On the other hand, in three cases, the manual cytospin was acellular, and in the matched automatic cytospins, 3 to 26 cells were counted. In all these cases, the TNCC on the Neubauer counting chamber was ≤5 cells/μL (no pleocytosis). 

In the cases with an increased TNCC, we were able to count 200 or more cells in the automatic and paired manual cytospin preparations in 14 cases. In two cases, the manual cytospin presented more than 200 cells, whilst in the automatic cytospin, the cell count at the microscope was between 150 and 200 cells. In one case of mild pleocytosis, the cell count in the automatic cytospin was 178 cells and in the manual cytospin was 192 cells. 

The kappa value for the cell counting in the cytospins, considering the categories 0, 1–199 cells and more than 200 cells, was 0.78 (95% CI, 0.64–0.93), indicating a substantial agreement between both methods. When cases with more than 200 cells in both cytospin smears were excluded, there was not a significant difference between the total number of cells yielded using automatic and manual cytocentrifugation. Similarly, the percentages of each cell type and of the non-diagnostic cells in each cytospin smear were not significantly different.

**Figure 2:**
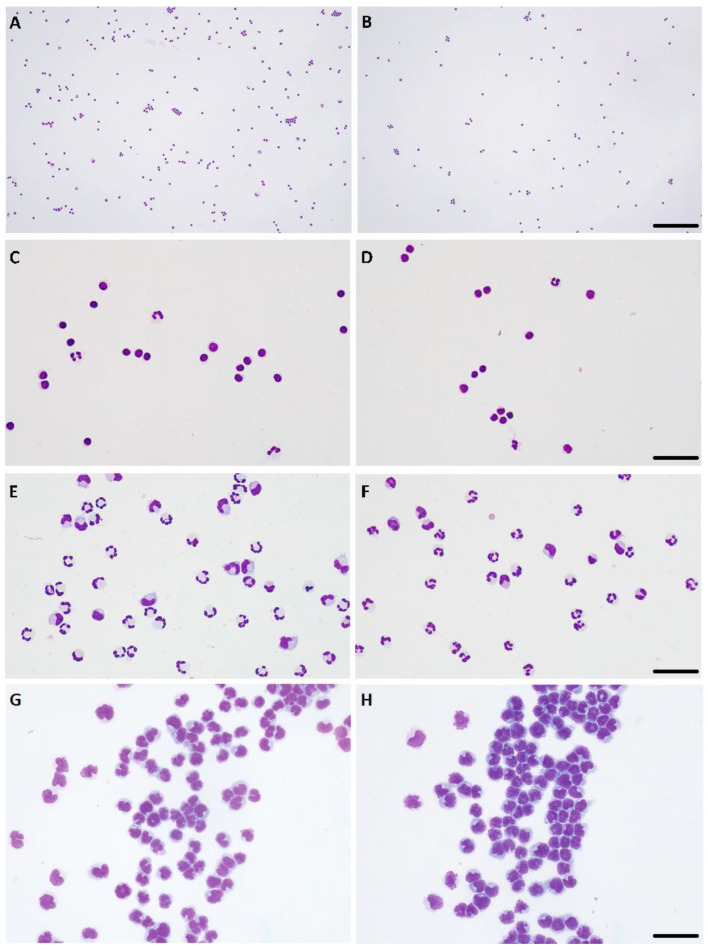
Paired cytospins generated using automated (**A**,**C**,**E**,**G**) and manual (**B**,**D**,**F**,**H**) cytocentrifugation. (**A**–**D**) Lymphocytic pleocytosis, ×10 objective, bar = 180 μm (**A,B**), ×40 objective, bar = 30 μm (**C**,**D**). (**E**,**F**) Mixed-cell pleocytosis, predominantly neutrophilic, ×40 objective, bar = 40 μm. (**G**,**H**) Lymphocytic neoplastic pleocytosis, compatible with lymphoma of the central nervous system, ×40 objective, bar = 40 μm. Hemacolor stain.

In normal CSF (i.e., without pleocytosis), the percentage of monocytoid cells, neutrophils and lymphocytes in the manual and automatic cytospins were moderately to strongly correlated (Spearman’s Rho values = 0.38, 0.52, 0.39; *p* = 0.02, 0.001, 0.02, respectively). In this type of CSF specimen, only the correlation of non-diagnostic cells was not statistically significant. When CSF presented increased cellularity (independently of the degree of pleocytosis), the Spearman’s Rho value was ≥0.93 (*p* < 0.0001), indicating a very strong to almost perfect correlation between the two cytocentrifugation methods in the percentages of monocytoid cells, neutrophils and lymphocytes. Only for the non-diagnostic cells the correlation was lower, with a Rho value of 0.68 (*p* = 0.004). These statistical results supported the very good agreement between the cytologic diagnoses of the type of pleocytosis made in each cytospin smear (Table 2, Figure 2).

### 3.4. Presence of Artifacts

The evaluation of artifacts (pseudopods and vacuoles) was only performed in the prospective cases; one of these samples was excluded because a large amount of blood in the background for both cytospins hampered the clear identification of such morphologic alterations. The presence of artifacts was noted in 41 out of the 47 smears obtained using automatic cytocentrifugation and 36 out 47 smears obtained using manual cytocentrifugation (Table 3). No significant differences regarding the presence of artifacts, either vacuoles or pseudopods, existed between the cytocentrifuge methods. 

### 3.5. Presence of RBCs

The presence of RBCs on the Neubauer counting chamber was detected in 30 cases. In the cytospins, the RBCs were present in 23 smears prepared automatically and 21 smears prepared manually (Table 4). 

The presence of RBCs on the cytological slide was moderately concordant [kappa 0.55 (95% CI 0.33–0.78)]. Consequently, the number of RBCs observed in each preparation was moderately correlated (Spearman’s Rho = 0.69; *p* < 0.0001).

## 4. Discussion

The most important adversities while undertaking a cytologic analysis of CSF are the cell concentration and the preservation of the cellular elements and of their morphologic features [19]. At most reference laboratories, automated cytocentrifuges that allow for rapid preparation of smears with good cytomorphologic preservation are used for preparing CSF cytologic specimens. However, in the majority of veterinary clinics, this type of cytocentrifuge is cost-prohibitive [9]. When comparing an automatic cytocentrifuge with sedimentation techniques, the first method costs significantly more, but it is also three times faster and yields much better results [20]. The time for analysis is, indeed, an important pitfall in CSF evaluation since it is a highly labile fluid and analysis should be performed immediately after collection. That being said, the average interval between sample collection and CSF analysis in a laboratory is 4 to 8 h; this time lapse can lead to potential sample deterioration and ultimately affect clinical diagnosis and treatment [21]

A comparison between a commercially available automatic cytocentrifuge and a manual converted salad spinner to concentrate low-cellularity fluids, including CSF specimens, has already been published [14]. This publication, however, included only a small number of CSF samples. The present prospective study was designed in order to compare the analysis of paired CSF smears following manual centrifugation with those of standard automatic cytocentrifugation in a large case series [22].

The manual cytocentrifugation method allowed for the preparation of rapid and low-cost, in-house cytospins of CSF [14]. The morphologic cell preservation was similar to that from cytospins obtained with the automated cytocentrifuge. Additionally, the presence of artifacts (both vacuolization and pseudopods) and non-diagnostic cells were comparable between the two methods. In addition to being lower in cost, the manual cytocentrifuge has reusable parts (cell concentrators and metallic clips) and inexpensive and readily available replacement parts (filters and glass slides) [14]. It should be stressed that using manual cytocentrifugation was also technically easy and repeatable in nature.

The recovery and the preservation of cells using the cytocentrifuge system have been reported to be unpredictable [23]. In a study in human medicine, where four cytospin slides were run simultaneously (with the same technique and similar amounts of CSF, from a single specimen), four different scenarios were produced, ranging from no cells to the presence of innumerous well-preserved cells distributed at the edges of the preparation [23]. This unpredictability may explain why there was a difference in cell counts in some paired cytospins from CSF specimens. In our study, this occurred more in CSF samples without pleocytosis. It should be noted that this *unpredictability* in the number of cells recovered has been reported in previous studies in CSF samples in dogs [18]. For instance, it has been reported that samples with ≤1 cell/μL on the hemacytometer may yield cytospins with 14 to 174 cells [18]. In relation to the RBCs, it was noted that in some cases they were counted in the hemocytometer, but were not present in the corresponding cytospins. However, none of these differences were found to be statistically significantly. 

One of the most relevant limitations of this study is the high proportion of cases with no pleocytosis (n = 38) (n = 17). It should be stressed that this proportion of altered CSF (around 30%) is within the range published in previous studies that validated the use of automated cell counters in CSF (% samples with pleocytosis: 18% [24] to 32% [6]). Similar to these other previous studies [6,8,24], we fulfilled the recommendations for method comparison of including more than 40 patient samples, representing the spectrum of diseases encountered in clinical practice [22]. 

## 5. Conclusions

A manual cytocentrifuge created from an inexpensive adapted salad spinner allowed for easy, affordable, repeatable and comparable CSF cytology results when compared to the standard automated cytocentrifuge. The availability of such a device for routine clinical use allows for more immediate processing of CSF samples, lessening the likelihood of sample deterioration during transport to an external laboratory for evaluation, ultimately improving patient diagnosis and potential treatment. 

## Figures and Tables

**Table 1:** Distribution of cases according to the total nucleated cell count performed in the manual hemocytometer (Neubauer counting chamber).

	Prospective Cases	Retrospective Cases	TOTAL
No pleocytosis	33	5	38
Pleocytosis	15	2	17
Mild	6	2	8
Moderate	5	0	5
Severe	4	0	4
TOTAL	48	7	55

**Table 2:** Cytologic classification of the type of pleocytosis according to the predominant cell type present [4] in automatic and manual cytocentrifugation smears.

Pleocytosis	Automatic	Manual
Mild		
Case n. 08	Mixed-cell	Mixed-cell, predominantly lymphocytic
Case n. 10	Monocytoid	Monocytoid
Case n. 12	Mixed-cell, predominantly lymphocytic	Mixed-cell, predominantly lymphocytic
Case n. 13	Mixed-cell, predominantly neutrophilic	Mixed-cell, predominantly neutrophilic
Case n. 21	Neutrophilic	Neutrophilic
Case n. 27	Lymphocytic	Lymphocytic
Case n. 30	Mixed-cell	Mixed cell
Case n. 44	Lymphocytic	Lymphocytic
Moderate		
Case n. 05	Neutrophilic	Neutrophilic
Case n. 15	Lymphocytic	Lymphocytic
Case n. 32	Mixed-cell, predominantly monocytoid	Mixed-cell
Case n. 41	Mixed-cell, predominantly lymphocytic	Mixed-cell, predominantly lymphocytic
Case n. 55	Lymphocytic	Lymphocytic
Severe		
Case n. 03	Lymphocytic	Lymphocytic
Case n. 24	Neutrophilic	Neutrophilic
Case n. 47	Lymphocytic	Lymphocytic
Case n. 54	Mixed-cell, predominantly monocytoid	Mixed-cell, predominantly monocytoid

**Table 3:** Contingency table comparing presence of artifacts in the 47 cases evaluated, according to the type of cytocentrifugation (automatic/manual).

MANUAL	AUTOMATIC			
		Presence		Total
	Presence	31	Presence	36
	Absence	10	Absence	11
	Total	41	Total	47

**Table 4:** Matrix comparing presence of red blood cells in the 55 cases, according to the type of cytocentrifugation (automatic/manual).

MANUAL	AUTOMATIC			
		Presence	Absence	Total
	Presence	17	4	21
	Absence	6	28	34
	Total	23	32	55

## Data Availability

The data generated or analyzed during this study are included in this published article. The raw data are available upon justified request to the corresponding author.
